# Expansive growth of two glioblastoma stem-like cell lines is mediated by bFGF and not by EGF

**DOI:** 10.2478/raon-2013-0063

**Published:** 2013-10-08

**Authors:** Neza Podergajs, Narve Brekka, Bernhard Radlwimmer, Christel Herold-Mende, Krishna M. Talasila, Katja Tiemann, Uros Rajcevic, Tamara T. Lah, Rolf Bjerkvig, Hrvoje Miletic

**Affiliations:** 1 Department of Genetic Toxicology and Cancer Biology, National Institute of Biology, Ljubljana, Slovenia; 2 Department of Biomedicine, University of Bergen, Bergen, Norway; 3 German Cancer Research Centre, Division of Molecular Genetics, Heidelberg, Germany; 4 Division of Neurosurgical Research, Department of Neurosurgery, University of Heidelberg, Heidelberg, Germany; 5 NorLux Neuro-Oncology Laboratory, Centre de Recherche Public de la Santé, Luxembourg; 6 Blood Transfusion Centre of Slovenia, Ljubljana, Slovenia; 7 Department of Biochemistry, Faculty of Chemistry and Chemical Engineering, University of Ljubljana, Ljubljana, Slovenia; 8 Department of Pathology, Haukeland University Hospital, Bergen, Norway

**Keywords:** glioblastoma, bFGF, EGF, EGFR, stem cell cultures

## Abstract

**Background:**

Patient-derived glioblastoma (GBM) stem-like cells (GSCs) represent a valuable model for basic and therapeutic research. GSCs are usually propagated in serum-free Neural Basal medium supplemented with bFGF and EGF. Yet, the exact influence of these growth factors on GSCs is still unclear. Recently it was suggested that GBM stem-like cells with amplified EGFR should be cultured in stem cell medium without EGF, as the presence of EGF induced rapid loss of EGFR amplification. However, patient biopsies are usually taken into culture before their genomic profiles are defined. Thus, an important question remains whether GBM cells without EGFR amplification also can be cultured in stem cell medium without EGF.

**Meterials and methods:**

To address this question, we used two heterogeneous glioblastoma GSC lines (NCH421k and NCH644) that lack EGFR amplification.

**Results:**

Although both cell lines showed very low EGFR expression under standard growth conditions, bFGF stimulation induced higher expression of EGFR in NCH644. In both cell lines, expression of the stem cell markers nestin and CD133 was higher upon stimulation with bFGF compared to EGF. Importantly, bFGF stimulated the growth of both cell lines, whereas EGF had no effect. We verified that the growth stimulation by bFGF was either mediated by proliferation (NCH421k) or resistance to apoptosis (NCH644).

**Conclusions:**

We demonstrate that GSC cultures without EGFR amplification can be maintained and expanded with bFGF, while the addition of EGF has no significant effect and therefore can be omitted.

## Introduction

Glioblastoma (GBM) is the most frequent and most malignant primary brain tumor. Despite improvement of surgical removal, chemotherapy and radiotherapy, the majority of patients live only less than 16 months after diagnosis.^1^ The hypothesis that so-called cancer stem-like cells (CSCs) are responsible for tumor development has gained considerable attention, albeit with controversial views.^2^,^3^ These cells may reflect characteristics of normal neural stem cells, such as self-renewal, expression of neural stem cell markers, and the capacity to differentiate into phenotypes resembling neuronal and glial cell lineages.^4^–^6^ It has been postulated that CSCs are responsible for tumor recurrence after treatment^7^, which makes them highly relevant targets. Although the presence of GBM stem-like cells (GSCs) in human tumors is debated^8^,^9^, GSCs cultured in Neurobasal (NB) medium supplemented with epidermal growth factor (EGF) and basic fibroblast growth factor (bFGF) currently represent the standard model in GSC research. The presence of EGF and bFGF in NB medium have been regarded as necessary supplements to maintain the stem cell-like features and also to stimulate their growth.^10^ Moreover, it has been shown that GSCs cultured under these conditions preserve the genetic profile and characteristics of the human tumor of origin much better than cells cultured in serum monolayer conditions.^11^,^12^ However, research is still needed to define optimal conditions for GSCs cultures.

In this respect, an open question remains about the influence of EGF and bFGF on GSC cultures and whether both of these growth factors are really required. Recently, Schulte *et al*.^13^ demonstrated that GBMs with EGF-receptor (EGFR) amplification are preserved much better in NB medium with bFGF, but without EGF. This observation is highly relevant since the tyrosine kinase receptor EGFR is often amplified or mutated in GBMs. About 40–50% of GBM patients show *EGFR* amplification and half of these harbor also the mutated EGFRvIII.^14^,^15^ Yet, over 50% of GBMs do not have amplifications or mutations of the EGFR. Thus, the optimal culture conditions irrespective of the genotype need to be defined in order to study important aspects of GBM biology within individual, genotypically preserved cultures.

In the present study, we investigated the impact of EGF and bFGF on GSCs without EGFR amplification in order to define whether the GSC culture conditions should be changed in general, as suggested by Schulte *et al*.^13^ We demonstrated that the presence of bFGF in the culture medium is crucial for GSC growth, whereas EGF had no significant stimulatory effects on these cells.

## Materials and methods

### Cell lines

Two GSC lines, NCH644 and NCH421k, which have been isolated as described previously^16^, were used in this study. The cells were grown as suspension spheroids in NB medium (Invitrogen, Life Tech., Grand Island, NY) containing 2 mM L-glutamine, 1× penicillin/streptomycin (both from Lonza, Basel, Switzerland), 1× B-27 (Invitrogen, Life Tech., Grand Island, NY) and 1 U/ml heparin (Sigma-Aldrich, Steinheim, Germany). 20ng/ml bFGF and/or EGF (both from PeproTech, Inc., Rocky Hill, NJ) were added to NB medium to create different growth conditions as used for the experiments described in this section. NB medium with addition of EGF and bFGF is referred to as complete medium. Before the start of experiments, the NCH644 and NCH421k cells were initially cultured for 7 and 10 passages in complete medium, respectively. Neurospheres were dissociated mechanically when they reached approximately 200 μm in diameter.

### Comparative genomic hybridization (CGH)

Both cell lines used for this experiment were grown in complete NB medium. Array-CGH profiling was performed as previously reported.^17^ Data were analyzed based on EnsEMBL (version 69) using packages of the Bioconductor project^18^ implemented in our in-house developed ChipYard framework for microarray data analysis (http://www.dkfz.de/genetics/ChipYard/). The array-CGH analyses of NCH421k had previously been published^11^ and were re-analysed for this study. The array-CGH analyses of NCH644 were performed exclusively for the present study.

### Flow cytometry

Before the FACS analysis, cells were grown for ten days in NB medium without growth factors. The cells were then treated with EGF and/or bFGF for 48h. The cells were washed with PBS, stained with an anti-EGFR antibody diluted in 1:50 ratio (Santa Cruz Biotechnology, Inc., Santa Cruz, CA) for 30 minutes at 4°C, washed twice with PBS and stained with secondary Alexa Fluor 488^®^ antibody diluted in 1: 200 ratio (Invitrogen, Life Tech., Grand Island, NY) for 30 minutes at 4°C. Finally, the cells were washed twice, resuspended in PBS, and analyzed by FACSCalibur^TM^ (BD Biosciences, San Jose, California).

### Western blotting

Prior to the experiment, cells were grown in complete NB medium. On the day of the experiment, the cells were divided into four groups and grown under different conditions for 14 days as stated in the results section. For protein isolation, spheroids were washed with PBS and lysed in lysis buffer (20 mM MOPS, pH 7.0, 2mM EGTA, 5mM EDTA, 30 mM NaF, 60 mM β-glycerophosphate, pH 7.2 20 mM sodium pyrophosphate, 1 mM sodium orthovanadate, 1 mM phenylmethylsulfonylfluoride, 3 mM benzamidine, 5 μM pepstatin A, 10 μM leupeptin, 1% Triton X-100, 1mM DTT). Proteins were quantified using Pierce BCA^™^ Protein Assay Kit (Pierce Biotechology, Rockford, IL). 20 μg of protein was loaded on NuPage^®^ Novex 4–12% Bis-Tris gels, run in MOPS running buffer (both from Invitrogen, Life Tech., Grand Island, NY) and blotted on nitrocellulose membrane (EMD Millipore, Massachusetts, USA). The nitrocellulose membrane was blocked for 1 hour at room temperature and incubated overnight at 4°C in buffer (PBS with 0.1% Tween 20 and 5% milk powder) containing the following primary antibodies: anti-nestin diluted in 1:1000 ratio (EMD Millipore, Massachusetts, USA), anti-CD133/1 clone AC133 diluted in 1:100 ratio (Miltenyi, Auburn, CA), anti-SOX2 diluted in 1:200 ratio (R&D Systems, Abingdon, UK), ant-EGFR diluted in 1:500 ratio (Santa Cruz Biotechnology, Inc., Santa Cruz, CA), and anti-GAPDH antibody diluted in 1:2500 ratio (Abcam, Cambridge, UK). Secondary antibodies anti-mouse IgG HRP diluted in 1:10000 ratio (Santa Cruz Biotechnology, Inc., Santa Cruz, CA) or anti-rabbit IgG HRP diluted in 1:2500 ratio (Promega, Fitchburg, Wisconsin) and WestFemto Chemiluminiscent Substrate (Pierce Biotechnology, Rockford, IL) were used for chemiluminescence detection. CD133, nestin and SOX2 expression results obtained from western blots were quantified using Image J software, version 1.46r (National Institutes of Health, http://rsbweb.nih.gov/ij/), following the developer’s instructions (http://rsb.info.nih.gov/ij/docs/index.html) for the analysis of the data. The quantification of results are expressed as relative protein expression.

### Cell growth assay

Cells were grown in NB medium without growth factors for ten days. 14 days before the start of the experiment, cells were divided into four groups and grown under different conditions as stated in the results section. On the starting day of the experiment, 1×10^4^ of cells were seeded onto T25 plates. Neurospheres were dissociated mechanically and counted every four days for the following twenty days under a light microscope (Olympus CKX31, Southend-on-Sea, UK) using 100× magnification.

### Cell cycle analysis

Cells, grown in NB medium without growth factors for ten days, were treated with EGF or bFGF and harvested 48 hours after the treatment. The preparation of the cells and cell cycle analysis were performed as described previously.^19^

### Apoptosis assay

Cells, grown in NB medium without growth factors for ten days, were treated with EGF or bFGF and harvested 48 hours after the treatment. The cells were washed with PBS and analyzed using Annexin V kit (Molecular Probes, Life Tech., Grand Island, NY) following the manufacturer’s instructions. Propidium iodide (PI; 50 μg/ml) and a FITC conjugated Annexin V protein were used for detection of apoptotic and necrotic cells by Accuri^®^ C6 Flow Cytometer (BD Biosciences, San Jose, California).

### Statistical analysis

One-way ANOVA test with Bonferroni post-hoc test was used to detect significant differences of groups. All statistical analyses were performed using GraphPad Prism version 5.01 for Windows (GraphPad Software, La Jolla, CA). P values < 0.05 were considered significant. Independent experiments were performed in triplicates.

## Results

### Stimulation of GSCs with bFGF increases expression of EGFR and stem cell markers

**FIGURE 1.**
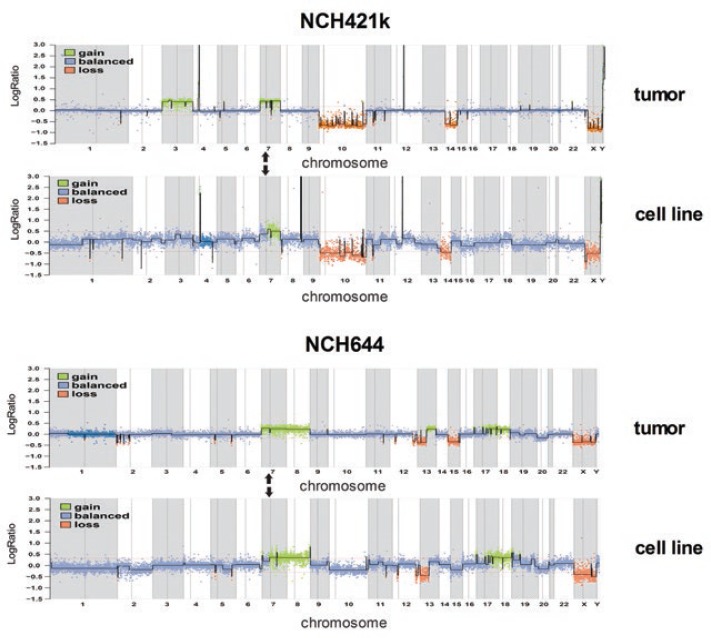
Array CGH profile of NCH421k and NCH644 cells and corresponding biopsies. Array-CGH analysis of NCH421k^11^ and NCH644 primary tumors and the respective cell lines showing typical GBM aberrations that are preserved in the cell lines, and consistent absence of EGFR amplification. Panels on the right show NCH644 primary tumor (top) and cell line (bottom). The relative DNA copy number of genome fragments are plotted on the Y-axis in order of their chromosome mapping positions (X-axis). Arrows mark the mapping position of the EGFR locus on chromosome 7p.

**FIGURE 2.**
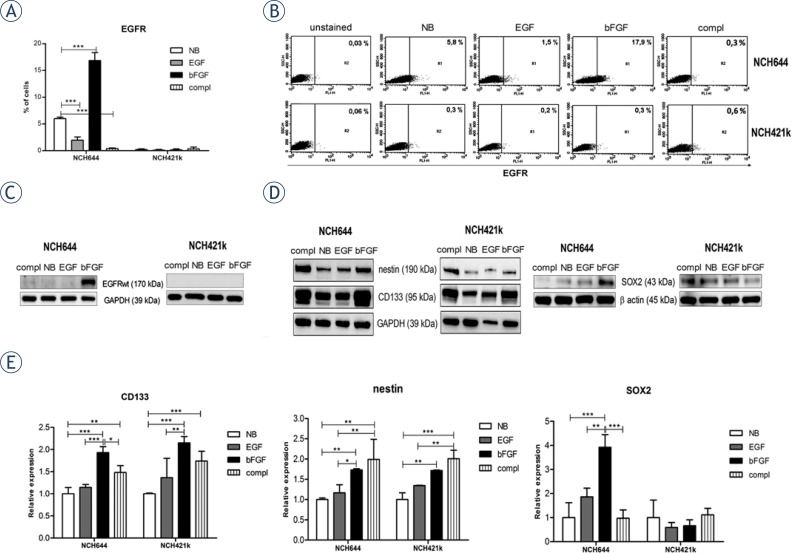
bFGF increases EGFR and stem cell marker expression in GSCs. **(A, B)** Flow cytometry analysis of EGFR expression in GSCs, grown in the presence of EGF and/or bFGF for 48 hours. bFGF alone increased the fraction of EGFR expressing cells in the NCH644 cell line, but not in the NCH421k, compared to medium without growth factors. Data shown are mean ± SD of three independent experiments, ***: p<0,001. **(C)** Western blots of GSCs cultured under different conditions. Presence of bFGF in culturing medium for 14 days upregulated the expression of EGFR in NCH644, but not in NCH421k. **(D)** Western blots of GSCs. Presence of bFGF in culturing medium for 14 days increased the expression of stem cell markers CD133 and nestin in both cell lines compared to unstimulated cells whereas the expression of SOX2 was increased only in NCH644 cells. EGF had no effect. **(E)** Quantification of western blots confirmed the significant higher expression of CD133 and nestin in both cell lines and a significant higher expression of SOX2 only in NCH644 cell line under bFGF stimulation. Data shown are mean ± SD of three independent experiments, *: p<0.05; **: p<0.01; ***: p<0.001. Labels: NB=Neurobasal medium without growth factors; compl=NB with EGF and bFGF; EGF=NB with EGF; bFGF=NB with bFGF.

In this study, we used two different, heterogeneous GSC lines, NCH421k and NCH644. These cell lines were derived from primary GBMs that showed typical GBM aberrations (*e.g*. loss of chromosome 10 and/or gain of chromosome 7) as indicated by DNA copy-number profiling by array-CGH. NCH421k additionally carried amplifications of the *PDGFRA* and *CDK4* gene loci. These copy number aberrations were well preserved in the cell lines (Figure 1). Both primary tumors as well as the NCH421k and NCH644 cell lines derived from them lack amplification of the *EGFR* gene locus (position indicated by arrows in Figure 1). Both cell lines belong to the proneural category according to the classification by Verhaak *et al*.^20^ (data not shown). Recently it has been shown that the presence of EGF in the culture medium leads to reduced EGFR expression in tumor cells with EGFR amplification.^13^ However, cells without EGFR amplification might also express EGFR. Therefore, we first determined the expression level of EGFR by flow cytometry in both cell lines under different growth conditions. Before the experiment, cells were cultured in NB medium without growth factors for 10 days and then divided into different groups for growth factor treatment as indicated in Figure 2. FACS analysis for EGFR revealed that 0.4 ± 0.14% of NCH644 cells and 0.35 ± 0.35% of NCH421k cells stimulated by both EGF and bFGF (complete NB medium) were positive. However, while stimulation with EGF alone, bFGF alone or medium without growth factors did not alter the expression of EGFR in NCH421k cells, there was a change in NCH644 cells under the defined conditions as shown in Figure 2A and B. bFGF stimulation for 48 hours substantially increased the fraction of EGFR expressing NCH644 cells to 16.85 ± 1.48% (p<0.001), while the absence of growth factors or EGF stimulation alone revealed only 6.00 ± 0.28% (p<0.001) or 1.95 ± 0.64% (p<0.001) EGFR expressing cells, respectively. Thus, bFGF stimulation alone had the strongest positive effect on EGFR expression, while complete medium containing both growth factors and EGF had a negative effect. The upregulation of EGFR in NCH644 was verified by western blot, where cells were stimulated with bFGF for 14 days (Figure 2C). The difference in response to bFGF between the two cell lines might be explained by the variable levels of EGFR that are initially present in GBMs without EGFR amplification. Only a fraction of GBMs without EGFR amplification naturally expresses EGFR.^21^

In addition, we investigated the impact of EGF and bFGF on stem cell marker expression, such as CD133, nestin and SOX2. Expression levels of CD133 and nestin were significantly increased in both cell lines upon bFGF stimulation whether alone or in complete medium whereas EGF alone did not affect stem cell marker expression (Figure 2D and E). In NCH644 cells only, SOX2 expression levels were significantly increased upon bFGF stimulation. These results show that bFGF alone as well as both growth factors in combination are able to maintain high stem cell marker expression in both GSC lines.

### bFGF, but not EGF, stimulates growth of GSCs

**FIGURE 3.**
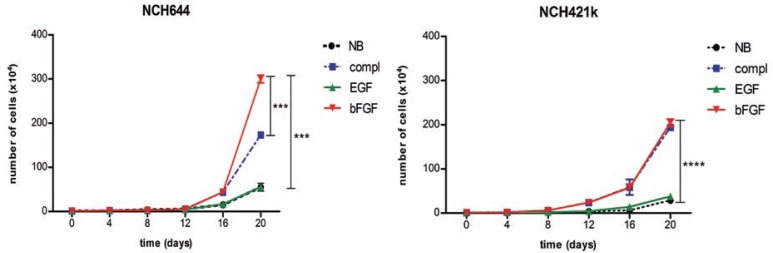
bFGF stimulates growth of GSCs. Growth curves of cells cultured in stem cell media differing in the presence of EGF and/or bFGF. Both cell lines showed the fastest growth upon bFGF stimulation, either alone or in complete medium with both growth factors whereas EGF alone did not significantly stimulate cell growth compared to medium without growth factors. Labels: NB=plain Neurobasal medium without growth factors; compl=NB with EGF and bFGF; EGF=NB with EGF; bFGF=NB with bFGF. Data shown are mean ± SD of three independent experiments with the following significant changes: ****: p<0,0001 for NCH421k (NB vs compl, NB vs bFGF, EGF vs compl, EGF vs bFGF); ***: p<0,001 for NCH644 (NB vs compl, NB vs bFGF, EGF vs compl, bFGF vs compl, EGF vs bFGF).

To define how bFGF and EGF affected the growth of the two cell lines, we cultured the cells in NB medium without growth factors for 10 days and then divided them into different groups for growth factor treatment as indicated in Figure 3. NCH421k and NCH644 cells formed spheroids under all conditions and there was no change in morphology of the cells as analyzed by light microscopy (data not shown). We generated growth curves over 20 days, which clearly showed that bFGF alone and complete medium increased growth of both cell lines by 5.5-fold (p<0.001) and 7.2-fold (p<0.001) for NCH644 and NCH421k, respectively (Figure 3), compared to EGF and medium without growth factors. Further, bFGF significantly increased growth of NCH644 cells over complete medium (p<0.001). Thus, bFGF appeared to be the most effective growth factor to culture and expand GSCs without EGFR amplification.

### GSCs proliferation and resistance to apoptosis are stimulated by bFGF

**FIGURE 4.**
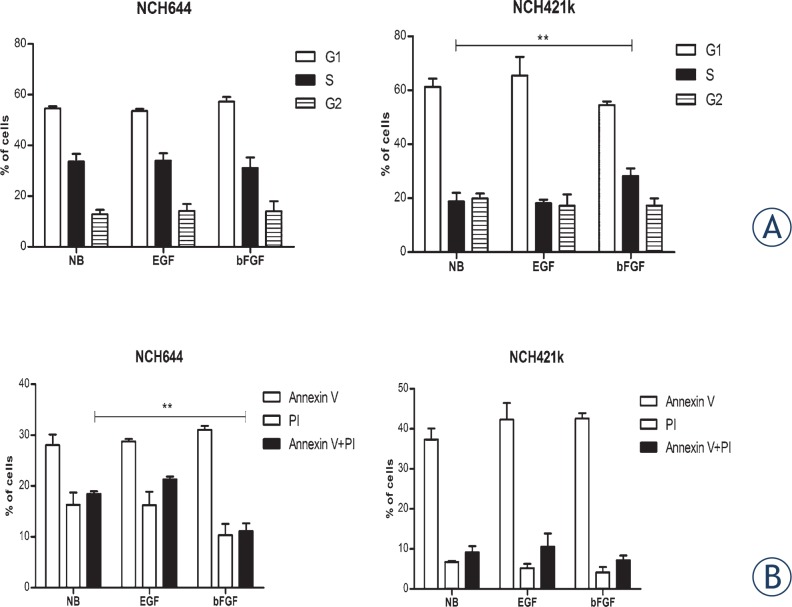
bFGF stimulates growth of GSCs due to increased viability or proliferation. **(A)** Cell cycle analysis of cells grown in the presence of EGF or bFGF. The relative changes in the cell number in G1, S and G2 phase were measured as indicators of cell proliferation. bFGF increased the number of cells in S phase in NCH421k cells, while there were no effects on the cell cycle in NCH644 cells. **(B)** Apoptosis assay of cells double labeled with Annexin V-FITC and PI. Percentages of cells in each histogram are representative of early apoptosis (labeled Annexin V), necrosis (labeled PI) and late apoptosis (labeled Annexin V+PI). Number of apoptotic NCH644 cells was decreased in presence of bFGF, but not in the presence of EGF. No significant changes upon EGF or bFGF stimulation were observed in the NCH421k cell line compared to the medium without growth factors. Labels: NB= Neurobasal medium without growth factors; compl=NB with EGF and bFGF; EGF=NB with EGF; bFGF=NB with bFGF. Data shown are mean ± SD of three independent experiments. **: p<0,01.

We then determined how bFGF and EGF affect cell cycle parameters and apoptosis. Cells were stimulated for 48 hours with bFGF or EGF. We observed that NCH421k cells showed a significant (p<0.01) increase in cells entering the S phase upon bFGF stimulation, verifying that the growth of NCH421k cells is stimulated by bFGF (Figure 4A). Stimulation with EGF had no effect on the cell cycle in NCH421k. Surprisingly, NCH644 cells did not show a significant difference upon stimulation with bFGF or EGF. Therefore, we used a cell death assay to analyze whether there is a difference in apoptosis. We observed 1.1–fold (p<0.01) less apoptotic NCH644 cells upon bFGF stimulation compared to unstimulated cells. EGF stimulation did not have this effect. In contrast to NCH644, the NCH421k cell line did not show any significant differences in the apoptosis assay (Figure 4B). Thus, in both GSC lines bFGF stimulated growth by either enhancing proliferation (NCH421k) or mediating resistance to apoptosis (NCH644), while EGF showed no effects.

## Discussion

An important prerequisite for culturing tumor cells from human biopsies is that the cultured cells should preserve the geno- and phenotype of the original tumor they were derived from. Culture media have an important impact as shown by Lee *et al*.^12^, where GBM derived stem-like cells cultured in serum-free medium with EGF and bFGF showed more similarities to the primary tumors compared to serum cultured cells. However, Schulte *et al*.^13^ reported that stem cell medium without EGF better preserved original tumor levels of EGFR amplification. Moreover, under high EGF concentrations, tumor cells did not retain EGFR amplification and lost tumorigenicity *in vivo*. This highlights the importance of the right choice of growth factors in serum-free culture media. In laboratory practice, human tumor biopsies are most often cultured before the genomic profile of the patient tumor is known. Thus, it would be an important advantage if culture conditions for EGFR non-amplified and EGFR amplified primary GBMs would be identical in order to preserve their characteristics and the original tumor genotype. GSCs in such cultures would thus represent a better model for studying the impact of different genetic abnormalities.

Here, we used two cancer stem-like cell lines, NCH644 and NCH421k, derived from patient GBMs without EGFR amplification, to define which growth factors are needed in the culture medium to maintain and expand GSCs. Importantly, we demonstrated that bFGF was the main growth stimulator of these GSC lines, while EGF did not have a significant effect. We verified that the cell growth effects of bFGF were mediated by an increased proliferation (NCH421k) or a decreased apoptotic rate (NCH644). This clearly shows that bFGF was able to induce expansion of GSC cultures in contrast to EGF. Investigating the effect of autocrine factors on glioblastoma growth, Kelly *et al*.^22^ claimed that glioma cells could also be effectively cultured without any growth factors as they have autocrine loops to stimulate proliferation. However, in our hands the GSCs without any growth factor stimulation were growing at a very slow rate, which is not sufficient for a considerable expansion of cells needed to effectively study GSCs.

When analyzing EGFR expression in the two GSC cell lines, we showed that only bFGF stimulation significantly increased the fraction of EGFR expressing cells compared to non-stimulated cells, whereas the presence of EGF alone or both growth factors in the culture medium significantly decreased this fraction. As this effect was observed in NCH644 only, the change in EGFR expression might also explain the significantly higher growth rate of NCH644 in bFGF only compared to complete medium, which was not observed in NCH421k. Upregulation of EGFR, which is a well characterized oncogene, might lead to a higher proliferation rate of cells. Thus, even in cells without EGFR amplification, EGFR expression can be better preserved when EGF is not present. This result was confirmed in a recent study by Mazzoleni *et al*.^23^ showing that reduction of EGF levels in culture media resulted even in re-expression of EGFR in previously negative cells. Taken together this may indicate either a transcriptional downregulation of the receptor induced by EGF or the presence of a negative regulatory feedback loop, activated upon excessive EGF binding, which may result in endocytosis and degradation of EGF-EGFR complexes.^24^ Although in cancer this loop may be normally bypassed in order to provide continuous oncogenic signaling, the chronic stimulation by EGF in stem cell media might result in an overstimulation of receptor activity, triggering its degradation. In contrast, upon bFGF stimulation, the increased EGFR expression which was observed in our experiments, may be induced by a cross-talk between EGFR and FGFR signaling pathways which often overlap.^25^ FGFR activation upon bFGF binding may stimulate similar signaling transducers as binding of EGF to EGFR. Therefore, it is possible that FGFR activation through bFGF induces a positive feedback loop to increase EGFR expression.

Investigating the effects of both growth factors on expression of GSC stem cells markers, we showed that bFGF stimulation increased the expression of CD133 and nestin, while EGF did not. This contradicts the findings by Soeda *et al*.^26^ showing that EGF increased CD133 expression in a dose dependent manner. However, the stem-like tumor cells used in their study expressed high levels of EGFR in contrast to the NCH644 and NCH421k cells. Further, long-term stimulation of EGFR amplified/highly expressing tumor cells with EGF would result in down-regulation of EGFR and in the worst case loss of tumorigenicity^13^, eliminating EGF as a candidate growth factor for long-term stimulation of EGFR expressing cells. The present work indicates that bFGF alone is sufficient to preserve stem cell markers in GSCs, such as CD133 and nestin, which is important in order to study characteristics of the GSC phenotype.

In summary, using two different GSC lines without EGFR amplification, we demonstrated significant stimulatory effects of bFGF on the expression of stem cell markers and expansion of these cells. In contrast, EGF did not have any measurable effect. Moreover, EGF seemed to down-regulate EGFR expression, similar as reported for EGFR amplified GSCs^13^,^23^, suggesting that GSCs should be cultured in stem cell medium supplemented with bFGF only to better preserve the original GSC genotype.
